# IUC22370-79 Sleep disturbances in androgen deprivation therapy (ADT)-treated patients: a narrative review

**DOI:** 10.1093/oncolo/oyaf248.047

**Published:** 2025-08-29

**Authors:** F Rajaee Rizi

**Affiliations:** Department of Urology, Al-Zahra Hospital, Isfahan University of Medical Sciences, Isfahan—Iran, Islamic Republic

## Abstract

**Background:**

Androgen deprivation therapy (ADT) is a primary treatment for prostate cancer, significantly affecting patients’ quality of life. Sleep disturbances are frequently reported among ADT-treated individuals, impacting both physical and mental well-being. This narrative review examines the prevalence, underlying mechanisms, management strategies, and future research directions related to sleep disturbances in ADT-treated patients.

**Methods:**

A systematic literature search was conducted using PubMed, Scopus, MEDLINE, and Cochrane Library. Eligible studies—including clinical trials, observational studies, meta-analyses, and systematic reviews—were selected based on predefined inclusion and exclusion criteria. Extracted data were synthesized into thematic categories focusing on prevalence, pathophysiology, management strategies, and treatment outcomes.

**Results:**

Sleep disturbances are common among ADT-treated patients, presenting with varying severity and functional impact. Underlying mechanisms include hormonal alterations, psychological factors, and comorbid conditions. Both pharmacological and non-pharmacological interventions—such as cognitive behavioral therapy for insomnia (CBT-I) and pharmacotherapy—show potential for symptom relief. However, standardized treatment protocols remain limited.

**Conclusions:**

Sleep disturbances in ADT-treated patients pose a significant clinical concern. Despite existing management strategies, further research is necessary to elucidate underlying mechanisms and establish tailored, evidence-based interventions. Routine assessment and management of sleep disturbances by clinicians are essential to enhance patient quality of life. Future studies should prioritize personalized treatment approaches and long-term outcomes.

